# Seasonal Blowfly Distribution and Abundance in Fragmented Landscapes. Is It Useful in Forensic Inference about Where a Corpse Has Been Decaying?

**DOI:** 10.1371/journal.pone.0099668

**Published:** 2014-06-11

**Authors:** Jabi Zabala, Beatriz Díaz, Marta I. Saloña-Bordas

**Affiliations:** 1 Department of Zoology and Animal Cell Biology, Faculty of Science and Technology (UPV/EHU), Bilbao, Spain; 2 Sebero Otxoa, 45, 5 B. 48480 Arrigorriaga, Biscay, Spain; 3 Department of Entomology, Aranzadi Science Society, San Sebastián, Guipúzcoa, Spain; Roehampton university, United Kingdom

## Abstract

Blowflies are insects of forensic interest as they may indicate characteristics of the environment where a body has been laying prior to the discovery. In order to estimate changes in community related to landscape and to assess if blowfly species can be used as indicators of the landscape where a corpse has been decaying, we studied the blowfly community and how it is affected by landscape in a 7,000 km^2^ region during a whole year. Using baited traps deployed monthly we collected 28,507 individuals of 10 calliphorid species, 7 of them well represented and distributed in the study area. Multiple Analysis of Variance found changes in abundance between seasons in the 7 analyzed species, and changes related to land use in 4 of them (*Calliphora vomitoria*, *Lucilia ampullacea*, *L*. *caesar* and *L*. *illustris*). Generalised Linear Model analyses of abundance of these species compared with landscape descriptors at different scales found only a clear significant relationship between summer abundance of *C. vomitoria* and distance to urban areas and degree of urbanisation. This relationship explained more deviance when considering the landscape composition at larger geographical scales (up to 2,500 m around sampling site). For the other species, no clear relationship between land uses and abundance was found, and therefore observed changes in their abundance patterns could be the result of other variables, probably small changes in temperature. Our results suggest that blowfly community composition cannot be used to infer in what kind of landscape a corpse has decayed, at least in highly fragmented habitats, the only exception being the summer abundance of *C*. *vomitoria*.

## Introduction

An adequate knowledge of necrophagous blowfly species ecology and geographical abundance has a direct application in forensic science, as well as in other fields of biomedical disciplines. This is due to their role as indicators for forensic studies, myiasis producers, and pathogen vectors [1], [2]. Species of the family Calliphoridae are currently the most commonly used in forensic research [1]. There are hitherto many studies on their ecology that compare abundances among seasons and/or habitat categories to detect forensically meaningful species and to help inference in applied research and criminal cases. However, this knowledge is usually restricted to some areas and the ecology of most species involved is poorly understood, especially with regard to what factors rule their distribution and abundance within the landscape. In this way, for instance, Martínez-Sanchez *et al*. found seasonal differences in the abundance of blowflies in pasture and forest areas in Spain [3], and Baz *et al.* found differences in the distribution and abundance of calliphorid species along altitudinal gradients [4]; Hwang and Tuner found spatio-temporal changes in necrophagous dipterans abundance in London area [5]; Brundage *et al.* analyzed changes in the carrion fly community of central California [1], and found that species composition differed through seasons and environments; and Arnaldos *et al.* in Spain [6], together with Kavazos and Wallman in South Eastern Australia [2], reported seasonal changes in abundance and habitat preferences for some species. All these results are encouraging and suggest the possibility that, in case of need, inference on where a corpse has been could be made on the basis of forensic entomology science.

Despite these findings, results are difficult to extrapolate to other areas for several reasons. One is that other geographical areas may harbor different communities with other forensic indicator species [7], [8]. A more important one is that most studies use simple and categorical habitat descriptors (i.e urban, rural, and natural) without taking into consideration internal variability that can hardly be applied in complex landscapes. In fact, most studies compare sampling points in rather homogenous patches embeddedin a mixed landscape, but paid little attention to how the mosaicism in landscape matrix might affect to the blowfly community and abundance of species.

Habitat use and selection result from several processes that take place at different scales. Johnson [9] defined four orders of habitat selection, ranging from the selection of a large geographical area to microhabitat selection. Nevertheless, studies on blowflies usually relied on the characteristics of the habitat at the sampling point, despite some blowflies may be dispersed beyond 2 km in a single day [10]. Finally, no study has yet analyzed what environmental variables within the habitat categories influence the abundance of blowfly species, and at what landscape scale.

Most of Western Europe makes a good instance of a complex landscape, with dense human population inhabiting urban areas scattered in a highly fragmented rural landscape of bocage, meadows, orchards, cultures, woods, forest cultures, small towns and hamlets, together with a dense network of transport infrastructures. Although some studies analyzed some gradients [3], [4], [5], no study has yet evaluated how the forensically significant fly community changes within such a landscape; which species are the most important or useful, and how are they affected by landscape composition at different scales. Therefore, we conducted a field survey of blowflies in Western Europe aimed to determine if there are fly species that have a potential use in forensic science to determine the landscape type in which a corpse has been decomposing. To determine it, we analysed: 1) the blowfly community in the area and test which are the potentially useful blowflies of the area in forensic science, if any, 2) what are the landscape variables influencing their presence, and 3) how do these variables and their importance change in different seasons and scales. We considered that, ideally, in order to be useful for forensic purpose, a blowfly species should: i) be abundant and well distributed in order to have good chances of being found in most cases, at least in a certain type of ecosystem; ii) show differences in abundance among seasons and land uses in order to be indicative of a certain kind of area; iii) its abundance correlated with the natural-urbanisation gradient, with the later explaining an important part of the variation in order to be a good indicator of urban, rural or natural areas, while avoiding biases due to other variables.

## Results

A total of 28507 adult calliphorids were captured representing ten different species (Table 1). Only adults were collected and considered in further analyses. *Calliphora vicina* and *C*. *vomitoria* were the most abundant species, with a total of 9883 and 6530 specimens captured year-round. *Lucilia caesar* and *L*. *ampullacea* were also abundant, represented with 5607 and 2225 specimens, although no individuals were detected in winter. The other six species where not so abundant and less than 1000 specimens were trapped year-round. However, three of these, *L*. *illustris*, *L*. *sericata* and *Chrysomya albiceps*, showed a marked seasonal abundance with a peak in summer, and we retained them for analyses in that season. Finally, three species, *L*. *cuprina*, *L*. *richardsi* and *L*. *silvarum,* were very rare and scarce, with less than 100 specimens collected year-round, therefore we regarded their presence as occasional and did not include them in the analyses.

**Table 1:** Captured califorid species and their seasonal abundance.

Species	*Calliphora vicina*				*Calliphora vomitoria*			
Season	Autumn	Winter	Spring	Summer	Autumn	Winter	Spring	Summer
**Captures**	94,75	183,14	462,76	262,89	14,38	91,63	418,19	93,49
**Positive locations**	57	54	58	58	45	36	48	42
**Species**	***Lucilia sericata***				***Chrysomya albiceps***			
**Season**	**Autumn**	**Winter**	**Spring**	**Summer**	**Autumn**	**Winter**	**Spring**	**Summer**
**Captures**	3,13	0,23	3,97	89,16	0,75	0	0,43	42,41
**Positive locations**	10	2	15	44	4	0	1	44
**Species**	***Lucilia caesar***				***Lucilia ampullacea***			
**Season**	**Autumn**	**Winter**	**Spring**	**Summer**	**Autumn**	**Winter**	**Spring**	**Summer**
**Captures**	51,50	0	99,05	487,47	41,75	0	45,95	163,61
**Positive locations**	41	0	41	56	38	0	39	50
**Species**	***Lucilia illustris***				***Lucilia cuprina***			
**Season**	**Autumn**	**Winter**	**Spring**	**Summer**	**Autumn**	**Winter**	**Spring**	**Summer**
**Captures**	3,25	0	3,10	29,40	0,13	0	1,21	5,54
**Positive locations**	8	0	12	39	1	0	4	11
**Species**	***Lucilia richardsi***				***Lucilia silvarum***			
**Season**	**Autumn**	**Winter**	**Spring**	**Summer**	**Autumn**	**Winter**	**Spring**	**Summer**
**Captures**	0,25	0	0,34	0,36	0	0	0,17	0,96
**Positive locations**	2	0	4	3	0	0	1	2

Captures indicates the average number of specimens collected per each day the traps were kept active in a given season, and Positive locations the number of different sampling units in which the species was found. For further detail: monthly species abundances in different areas, details on spatial distribution in Saloña *et al.* [12].

In general, blowflies were most abundant during Spring (12008 specimens captured; 34.5 specimens per trapping day) and Summer (9755 specimens; 36.8 per trapping day) than during Autumn (1679 specimens; 7.0 per trapping day) or Winter (2365 specimens; 9.2 per trapping day). Similarly, summer was the most diverse season and the one with most species (10 species, Shannon diversity index, S = 1.62), followed by Autumn and Spring (9 and 10 species, and S = 1.17 and S = 1.14 respectively). Winter was the season with lowest abundance of specimens, less species (just three, one of which only represented by two individuals), and smallest diversity values (S = 0.64).

Using one year-round data and the three habitat categories assigned in the field, a two way MANOVA was applied to check the influence of land use and/or season on the abundance of the seven tested species (Table 2). The MANOVA found statistically significant differences in the composition of blowfly communities among seasons and land uses, as well as in the interaction of the two categories. Species by species inspection of the results revealed that all the seven species showed significant seasonal variations in their abundance, while only *C*. *vomitoria*, *L*. *ampullacea*, *L*. *caesar* and *L*. *illustris* showed significant variation related to different Land uses. Finally, only *C*. *vomitoria*, *L*. *illustris* and *L. caesar* showed significant variations on abundance related to the interaction of Land use and season. These results suggest that all the seven species can be of forensic interest to assess about season, and four of them to elucidate issues related to Land use. Moreover, *C*. *vomitoria*, *L*. *illustris* and *L. caesar* appear to be potentially the most useful ones since their abundance is also related to the interaction of both variables.

**Table 2:** Results of the two-way MANOVA examining for effects of Land Use and Season on the abundance of selected species.

	Land use		Season		Land use×Season	
Global results	F value	*p*	F value	*p*	F value	*p*
	3.452	0.001	10.483	0.001	2.180	0.001
Results by species	*F* value	*p*	*F* value	*p*	*F* value	*p*
***C. vomitoria***	**9.988**	**<0.001**	**7.912**	**<0.001**	**3.938**	**<0.001**
*C. vicina*	0.278	0.758	**29.357**	**<0.001**	1.619	0.143
***L. caesar***	**5.809**	**<0.003**	**25.272**	**<0.001**	2.612	0.018
***L. ampullacea***	**4.473**	**<0.013**	**15.885**	**<0.001**	0.741	0.613
***L. illustris***	**9.389**	**<0.001**	**20.963**	**<0.001**	**5.511**	**<0.001**
*L. sericata*	0.296	0.7441	**13.671**	**<0.001**	0.284	0.944
*C. albiceps*	0.593	0.553	**24.933**	**<0.001**	0.522	0.791

Results of the global analysis as well as results of individual species are shown. We show the value of the statistic F and the p value for each case, global and specific, for the effect of Land use, Season and the mixed effect of Land use and Season.

In order to assess relationships between species’ abundance and land use variables and other landscape descriptors, the degree of correlation among species abundance was firstly analysed (Table 3). No strong negative correlations were found, i. e. adults of no species appear to suppress competitively any other adult specimens. Therefore, we analysed relationships between landscape at different scales and blowfly abundance using Generalized Linear Models.

**Table 3:** Seasonal correlation among blowfly species.

Spring	*C. vicina*	*L. ampullacea*	*L. caesar*			
*C. vomitoria*	0.526**	0.346**	0.650**			
*C. vicina*		0.277*				
*L. caesar*	0.425*	0.303*				
**Summer**	***C. vicina***	***L. ampullacea***	***L. caesar***	***Ch. albiceps***	***L. sericata***	***L. illustris***
*C. vomitoria*	−0.078	0.076	0.291*	0.216	−0.180	−0.088
*L. illustris*	0.371*	0.514**	0.187	0.394*	0.235	
*L. sericata*	0.331*	−0.174	−0.127	0.186		
*Ch. albiceps*	0.247	0.111	0.341*			
*L. caesar*	−0.094	0.630**				
*L. ampullacea*	−0.037					
**Autumn**	***C. vicina***	***L. ampullacea***	***L. caesar***			
*C. vomitoria*	0.444**	0.142	0.215			
*C. vicina*		0.318*	0.421*			
*L. caesar*		0.825**				
**Winter**	***C. vicina***					
*C. vomitoria*	0.518**					

We show the Pearson’s product-moment correlation coefficient, ranging from −1 (strong negative correlation) to +1 (strong positive correlation). Statistical significance of the correlations is shown with an asterisk (*) when p<0.05, and with two (**) when p<0.001.

For this purpose, those of the original 60 sites that were less than 200 metres apart from each other were discarded to avoid spatial pseudoreplication, as well as any traps in the same vegetation patch, those for which we got no information, or only partial information, about land uses in the area (digital maps were only available for the Basque Autonomous Region and some neighbouring areas, but in some instances the sampling points were close to borders of regions with no information). Therefore, only results from 55 sampling sites have been used in these analyses. The degree of correlation of predictor variables at different scales (100, 500 and 2500 m) was analyzed to aid in the interpretation of results (See Table S1). The area covered by forest was strongly correlated to the distance to the nearest dense urban area at the three scales. This is logical, since forested areas tend to be further from urban areas than crops or similar land uses. However, it was kept in the analyses because its relationship with urban was variable (Table S1). Altitude and Y UTM were also strongly correlated at the three scales, as a result of the orography of the region where altitude values grow north to south, from the coast to the Iberian plateau. Both values were kept for the analysis because associated to the Y UTM value there might be other climatic features that might affect fly abundance too (i. e. climatic and vegetation transition from Eurosiberian to Mediterranean). Finally, there were varying degrees of negative correlation among the area covered by different land uses (urban, rural and forest) at different scales (Table S1). This was not unexpected, since the total surface was constant at each scale, and large values of a given land use implied low values of the others.

When performing GLMs, inspection of the dispersion parameters of the models and their relation to the degrees of freedom suggested overdispersion in every case, and, in consequence, quasi-Poisson error structures were used [11]. Results of the GLMs for *Calliphora vomitoria* (Table 4) showed a more or less constant pattern of urban areas avoidance. This pattern is clear at every analysed scale during summer, when *C*. *vomitoria* was significantly more abundant at points far from urban areas. Moreover, these summer models were fair good and explained in every case more than 65% of the variation. At several scale-season interactions there was also a correlation between abundance and fragmentation, and in seasons other than summer, its abundance was related to geographical coordinates and landscape descriptors, but the relation was regarded “unclear” because the value of the scale parameter was close to zero (<0.001) (several significant relationships with a plain effect on abundance; Table 4).

**Table 4:** Results of GLMs analyzing relationships between considered variables at different scales and seasons with abundance of *C. vomitoria*.

	*Calliphora vomitoria*											
Season	Spring			Summer			Autumn			Winter		
Scale	100 m	500 m	2500 m	100 m	500 m	2500 m	100 m	500 m	2500 m	100 m	500 m	2500 m
Forest	−	Unclear(*)	Unclear(*)	+	−	Unclear	−	Unclear	Unclear	−	Unclear(*)	Unclear
Rural	−	Unclear	Unclear(*)	+	Unclear(*)	Unclear	Unclear	Unclear	Unclear	−	Unclear	Unclear
Urban	−	Unclear	Unclear(*)	+	Unclear(*)	Unclear	Unclear	Unclear	Unclear	−	Unclear	Unclear(*)
Altitude	+	−	−	+	+	+	+	+	+	−(*)	−	−
Y UTM	Unclear	Unclear	Unclear	Unclear	Unclear	Unclear	**+**(*)	Unclear	−Unclear	−	Unclear	Unclear
X UTM	Unclear	Unclear	Unclear(*)	Unclear	Unclear	Unclear	Unclear	Unclear	Unclear	−	Unclear	Unclear
Fragmentation	−	−	+(*)	+	**+**(*)	+	+	+(*)	+(*)	−	−	+
Dist. to Urban	+	+	+(*)	**+**	**+**(*)	**+**(*)	Unclear	Unclear	Unclear	+	Unclear	+
% Explained dev.	30.6	37.4	51.2	63.6	76.1	70.7	27.6	36.7	52.0	32.1	46.1	31.3

The sense of the relationships is shown with + in case of positive relationships and – for negative relationships (i.e. lower abundance with high values for the variable). When the regression was almost flat (scale parameter value<±0.001), we considered it unclear. Statistically significant relationships are shown with an asterisk (*), and the deviance explained in each case is shown in bottom row (in percentage). We used n = 55 in the 100 m scale, n = 50 in 500, and n = 36 in 2500 m.

The case of *L*. *caesar* was quite different. In most of the GLMs, its abundance was not related to any variable, whilst in some it was significantly related to geographic coordinates or altitude (Table 5). The only exceptions are the spring abundance at the 100 m scale, significantly related to fragmentation; and the autumn GLM at the 500 m scale, which found a significant relationship with the area covered by rural land uses (Table 5). The later however could be regarded as unclear as a consequence of the almost flat slope of the line (<0.001). Results for *L*. *ampullacea* (Table 6) are similar to those for *L. caesar*. Abundance of *L. ampullacea* at the 100 and 500 m scales was significantly related to either altitude or geographic coordinates in two of the three seasons analysed. Contrastingly, this effect disappeared at the 2500 m scale where no significant relationship was found. In both cases *L*. *caesar* and *L*. *ampullacea*, the GLMs explained only a small part of the variability in the data, less than 50% in every case but in two of the models. Finally, in the instance of *L*. *illustris* we only had enough data to conduct analyses for the summer period, when no significant relationships were found at any scale with the considered predictor variables (Table 7). In addition, models performed very poor at the three scales in terms of explained variance.

**Table 5:** Results of GLMs analyzing relationships between considered variables at different scales and seasons with abundance of *L. caesar*.

	*Lucilia caesar*								
Season	Spring			Summer			Autumn		
Scale	100 m	500 m	2500 m	100 m	500 m	2500 m	100 m	500 m	2500 m
Forest	+	Unclear	Unclear	+	Unclear	Unclear	+	Unclear	Unclear
Rural	+	Unclear	Unclear	+	Unclear	Unclear	+	Unclear(*)	Unclear
Urban	+	Unclear	Unclear	Unclear	Unclear	Unclear	+	Unclear	Unclear
Altitude	+	−	+	−	−(*)	−	+	+	+
Y UTM	Unclear	Unclear	Unclear	Unclear	Unclear	Unclear	**+**(*)	Unclear(*)	Unclear
X UTM	Unclear	Unclear	Unclear	Unclear	Unclear	Unclear	Unclear	Unclear	Unclear
Fragmentation	−(*)	−	+	+	+	+	+	−	+
Dist. to Urban	−	+	−	+	+	Unclear	−	−	−
% Explained dev.	35.1	33.9	23.5	29.4	438	3.0.4	43.7	59.3	58.0

The sense of the relationships is shown with + in case of positive relationships and – for negative relationships (i.e. lower abundance with high values for the variable). When the regression was almost flat (scale parameter value<±0.001), we considered it unclear. Statistically significant relationships are shown with an asterisk (*), and the deviance explained in each case is shown in bottom row (in percentage). We used n = 55 in the 100 m scale, n = 50 in 500, and n = 36 in 2500 m.

**Table 6:** Results of GLMs analyzing relationships between considered variables at different scales and seasons with abundance of *L. ampullacea*.

	*Lucilia ampullacea*								
Season	Spring			Summer			Autumn		
Scale	100 m	500 m	2500 m	100 m	500 m	2500 m	100 m	500 m	2500 m
Forest	+	Unclear	Unclear	+	Unclear	Unclear	+	Unclear	Unclear
Rural	+	Unclear	Unclear	+	Unclear	Unclear	+	Unclear	Unclear
Urban	+	Unclear	Unclear	+	Unclear	Unclear	+	Unclear	Unclear
Altitude	−(*)	−(*)	−	−	−	+	−	Unclear	−
Y UTM	Unclear	Unclear	Unclear	Unclear	Unclear	Unclear	**+**(*)	Unclear(*)	Unclear
X UTM	Unclear	Unclear	Unclear	Unclear	Unclear	Unclear	Unclear	Unclear	Unclear
Fragmentation	−	−(*)	+	+	+	+	+	−	+
Dist. to Urban	+	−	+	Unclear	+	Unclear	−	−	Unclear
% Explained dev.	36.4	46.0	36.8	34.0	37.4	44.5	37.1	39.8	49.6

The sense of the relationships is shown with + in case of positive relationships and – for negative relationships (i.e. lower abundance with high values for the variable). When the regression was almost flat (scale parameter value<±0.001), we considered it unclear. Statistically significant relationships are shown with an asterisk (*), and the deviance explained in each case is shown in bottom row (in percentage). We used n = 55 in the 100 m scale, n = 50 in 500, and n = 36 in 2500 m.

**Table 7:** Results of GLMs analyzing relationships between considered variables at different scales in summer with abundance of *L. illustris*.

	*Lucilia illustris*		
Season	Summer		
Scale	100 m	500 m	2500 m
Forest	−	Unclear	Unclear
Rural	−	Unclear	Unclear
Urban	−	Unclear	Unclear
Altitude	−	Unclear	+
Y UTM	Unclear	Unclear	Unclear
X UTM	Unclear	Unclear	Unclear
Fragmentation	−	+	+
Dist. to Urban	Unclear	Unclear	−
% Explained dev.	12.5	7.4	23.8

The sense of the relationships is shown with + in case of positive relationships and – for negative relationships (i.e. lower abundance with high values for the variable). When the regression was almost flat (scale parameter value<±0.001), we considered it unclear. The deviance explained in each case is shown in bottom row (in percentage). We used n = 55 in the 100 m scale, n = 50 in 500, and n = 36 in 2500 m.

The amount of explained variance increased as wide areas are considered. Generalized linear models at the 100 m scale explained an average of 34.7% (SD = 12.33) of the variance; at 500 m, they explained an average of 42.1% (SD = 16.83), and 42.9% (SD = 15.06) of the variance at the 2500 m scale.

## Discussion

Calliphorids were abundant in our study site. Species richness, with seven well represented species and three rare ones, was similar to that of previous studies; for instance, 5 spp. in Salamanca [3]; 7 spp in Madrid [12], 8 spp in Aragón [13], 11 spp in Portugal [14], 7 spp in California [1], 16 in Australia [2], among others. Blowfly abundance was higher in summer and spring than in other seasons, and only *Calliphora* species were abundant throughout the year. This result is in concordance with previous studies that found *Calliphora vicina* and *C*. *vomitoria* year-round while *Lucilia* species and *Ch*. *albiceps* were abundant only in summer [3], [15]. This is apparently due to the thermophilic nature of the later species [3].

The fact that there were no strong negative correlations between different species (Table 3) suggests that there is no competitive exclusion among adults of different species, or at least the existence of enough resources to allow a spatio-temporal coexistence. On the other hand, strong positive correlations of abundance are likely the result of favourable environmental conditions (temperature, humidity, shelter) for blowflies in the area.

Considering the observed differences in seasonal abundance/year-round abundance of blowflies, it is not surprising that the Multiple Analysis of Variance found significant changes in the seasonal abundance of all the species. In the same way, the effect of “habitat” or land uses in the abundance of certain species is also a well established fact [1], [5]. In our case the MANOVA selected only 4 species whose abundance was significantly related to land use in the sampling point. In the case of *C*. *vomitoria* (Table 4), the species showed a pattern of urban rejection that reached statistical significance during summer at every scale and in spring at the 2500 m scale. Furthermore, the summer models explained an important amount of the variance (up to 75%). At the 500 m scale, *C. vomitoria* also showed higher abundance in areas with dense forest cover, that is in concordance with an avoidance of urban areas, since the variable “forest” is negatively correlated with “urban” and “rural” (Table S1) in our area, however, the scale parameter of these correlations were very low, suggesting scarce effect on abundance. At the 2500 m scale, we found a tendency for urban rejection in winter and spring, which became unclear in autumn. Anyway, these models explained little variance, suggesting that the key variables ruling the model are not included in the analyses. The spring model produced unclear correlations with every land use, and a significant positive effect of fragmented landscape far from urban areas on abundance of *C. vomitoria*. Interestingly, winter models showed at every scale a negative relationship with altitude, that was significant at the 100 m scale; and autumn, at the 100 m scale, a positive one with Y UTM which in turn is strongly negative related to altitude (Table S1). This suggests that in autumn-winter, *C*. *vomitoria* is more abundant in the lowlands and in the north of the study area, where altitudes are lower and the coast has a warming effect on environmental temperature. Therefore, our results show that *C*. *vomitoria* is strongly negative related with urban areas in summer and this single variable explains an important part of variance of the data, while during the rest of the year this pattern does not hold, and in cold months abundance is related to thermal shelter areas. This effect of warm areas in cold months could partly explain the spring model at the 2500 m scale. In that case, areas around sampling points near the coast are sea water and therefore were not included in the model, so this higher abundance in warmer coastal areas results in an apparent avoidance of all the considered land uses. Nevertheless, Grassberger & Frank stated that *C. vomitoria* outnumber the other blowflies in an experiment done under controlled conditions in central Viena [16]. Taking under consideration the vegetation in their research area (a restricted forested backyard adequately preserved with thick underbrush) [16], we should not ignore the existence of adequate environment conditions for the presence of this species in otherwise “urban conditions”. This is in agreement with our findings: that using simple categories as descriptors of complex landscapes can have misleading results. Furthermore, the fact that in our models abundance during summer was related to distance to urban areas and not to land covered by urban or other uses suggests an avoidance pattern. *C. vomitoria* were abundant far from urbanised spots but its abundance was not affected by the amount of land devoted to urban uses. The reason for it remains unknown. On the other hand, other research has shown that the soil type and environmental conditions can affect the development of larvae, and that if the environment of the burial area is not considered researchers can incur in important errors in PMI estimation [17]. For instance, that study [17] found *C. vomitoria* on a 105-day-old corpse as a consequence of cold weather and particular burial conditions. In the instances of *L*. *ampullacea* and *L*. *caesar*, a statistically significant relationship was found with altitude and/or geographic coordinates, with three exceptions (Tables 5 and 6). In the same way, all these models explained low percentages of deviance. The pattern emerging from the model was similar for this two species: the abundance was higher in low altitudes and high Y UTM values (lower, northern areas). This altitude-geographic pattern and low explained variance suggest correlation with some not included variables, most likely temperature. Indeed, both species are known to be thermophilous [3], and have been previously reported from localities close to the coast in the study area and nearby ones [15], [18]. Regarding *L. caesar*, there are two season-scale combinations whose results differ from that pattern. On the first hand, the unclear (slightly positive) effect of rural areas on abundance in autumn at the 500 m scale is difficult to explain; a possible explanation could be higher availability of food and/or warmer refuges [5] in cattle rearing areas here defined as rural. In addition, the model also selected altitude as a significantly related variable. On the other hand, the negative effect of fragmentation on abundance in Spring at the 100 m scale suggest that the species is more abundant on simpler landscapes, which again is difficult to explain with the scanty knowledge on the ecology of the species. In the instance of *L. ampullacea* there is also a negative effect of fragmentation on abundance in spring, at the 500 m scale in this case, whose possible explanation again cannot be grasped.

Only during summer months, we captured enough specimens of *L. illustris* to conduct analyses, and GLMs found no significant relationships with any variable at any scale. Therefore, variables ruling abundance of *L. illustris* were not among the variables considered in our models, and not even strongly correlated to them.

Therefore, in spite of the fact that all the species analysed show seasonal statistically significant changes in abundance, three out of seven common species, *L. sericata*, *L. illustris* and *Ch. albiceps* are clear indicators of summer in forensic analyses. *C. vicina* and *C. vomitoria* are common year round with maximum abundances in spring time, whereas *L. caesar* and *L ampullacea* can be found through most of the year, with maximum abundance in summer. Regarding land use, the MANOVA identified 4 species that show different changes in abundance, but, after checking that result with individual anovas, the only species that can be considered as a clear indicator of it is *C. vomitoria* during summer, when it showed statistically significant changes in abundance with distance to urban areas, and the models explained important amounts of the observed variance. The negative effect of urban areas, or its equivalent positive effect of rural and forest areas, seemed to be important in some models of *C. vomitoria* for other seasons too, but they explained little variance. Furthermore, winter models clearly showed a switch in predictor factors for low altitudes in cold months, which are interpreted as an indirect effect of temperature. Concerning the other three species that showed significant changes among land uses in the MANOVA (Table 2), GLM results show that this difference is not related to land use itself but mainly to altitude and geographic coordinates, which somehow are correlated to land uses. This is logic, since variations in climate and temperature influence on soil productivity and, therefore, on main land uses.

There is broad evidence in literature of the thermophilic character of many blowfly species, including the species considered in the present study but the two *Calliphora* species [3], [5]. Our results suggest that differences in abundance among land uses detected by the MANOVA truly reflect differences in temperature correlated to it. A correlation with temperature, which was not possible to be measured in *situ*, may also explain the low percentages of explained variance. Furthermore, this could probably be the underlying cause for differences in blowfly communities in different land uses detected by other studies [1], [3], [5], [19]. Another factor correlated to land use that might affect abundance of blowflies, is local availability of carcasses and other food sources. Forested areas probably had more carcasses of small to large wild animals available, whereas in urban areas garbage and small carcasses might be the main resource. Rural areas probably are intermediate between the other two. The total abundance of food and its characteristics in each area type remains an unexplored issue, despite being a probable source of variability in blowfly community species composition and abundance.

Analysis of the amount of deviance explained at different scales suggest that considering land uses within large (>2000 m) distances around sampling points explains part of the variation in the data. This is probably related to the long dispersal capability of blowflies [10], and remarks the need to be careful when relating blowfly communities to a particular sampling point characteristics and/or making inferences on the area where a corpse has been found or decayed, considering only surrounding vegetation and land uses in small areas. The fact that explained deviance grew constantly with distance, suggests that abundance of different blowfly species could be affected by landscape structure at even larger distances than those considered in this study. A note of caution is required here since our results are preliminary, and our study was not primarily designed to test this particular point. Notwithstanding, further research is needed on this point, and can be useful for the interpretation of future forensic cases.

Our results have two important implications. One is that forensic inference should be drawn from local studies. Any inferences extrapolated from other studies must be considered with extreme care because communities vary, as has been found in other areas [1], [5], [20]. Another is that blowfly community composition cannot be used to infer in what kind of landscape a corpse has decayed, at least in complex and heterogeneous areas like Western Europe and other densely populated areas. The only exception to this point would be the summer abundance of *C*. *vomitoria* related to the urban-non urban gradient. This species might be of outmost importance in forensic research due to its avoidance of hard urban areas and its widespread distribution too.

In order to be able to relate blowfly abundance to landscape with forensic purposes, further research is needed, especially focused on the reliability of using results from other areas (either similar or nearby) in forensic cases. In addition, research on autoecology (temperature ranges, habitat use and selection, abundance and dispersion, and food preferences) of forensically important blowfly species is necessary to enlighten the interpretation of their presence and abundance in forensic cases.

## Materials and Methods

### Study Area

The study was conducted in the Basque Country (North of Spain), which is an area of about 7000 km^2^ of contrasting landscapes. Three mountain ridges run east-west across the territory, with their north aspects catching the humid winds from the Gulf of Biscay and eliciting rain. The north area, by the Gulf of Biscay, is warm, rainy and rugged with altitudes ranging from 0 to some 1500 m above sea level. In general terms, it has a humid temperate climate without dry season, and an average rainfall of 1200–2000 mm. [21], [22]. Valley bottoms are densely populated with villages, industrial areas and hamlets scattered in the landscape of meadows, and woodlots. Slopes are covered with forests and half of the surface is occupied by exotic tree cultures (mostly *Pinus radiata* and *Eucalyptus* spp). Highlands are typically meadows and pastures. The central area is a high plateau (some 600 m a.s.l., with an average rainfall of 750–900 mm.) mainly devoted to crops, with some scattered woods, and ranges with forested slopes. Climate there is Atlantic with a neat Mediterranean influence. The southernmost area lies by the Ebro river valley and it is mainly devoted to vineyards. Forests are scarce, occupying mainly mountain slopes and hilltops, and dominant species are evergreen oaks (*Quercus rotundifolia*) and Scottish pine (*P. sylvestris*) in some areas. Climate there is cold in winter and hot and dry in summer, with rainfall ranging from 500 to 1000 mm per year and taking place mainly in winter-spring. Northern valleys tend to be warmer in winter than southern ones, due to their lower altitudes and the proximity of the ocean.

**Figure 1:**
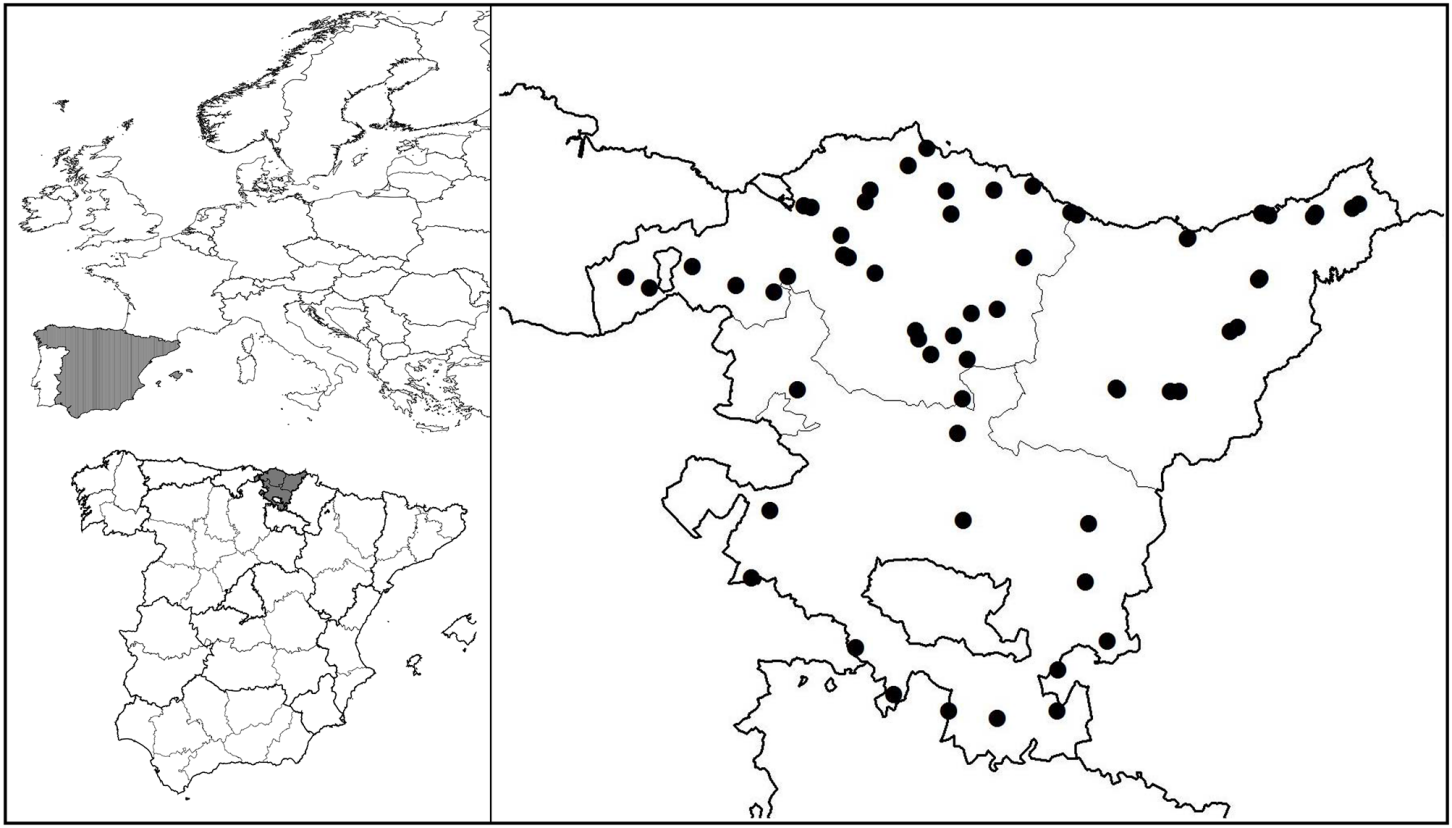
Study area location and sampling point distribution within it.

60 sites were sampled, more or less randomly distributed in the study area (Figure 1). A detailed reference to the specific samples used in this study has been previously described [22]. No natural areas or private farms were invaded as traps were placed in borders and rural pathways inaccessible to pets, cattle or humans, and properly labeled reporting the activity. Therefore, no specific permissions were required for these locations, as the field studies did not involve endangered or protected species [23] and all the activities described abided by spanish regulation and international ethical standards. To ensure that samples represent different uses and landscapes in heterogeneous areas, we placed the traps in pairs, one in an urban sampling point (city or village) and another in a nearby less altered one (rural or forest area). Following common forensic entomological practice we considered three environments: urban, rural, and forest [1]–[6] Traps were kept active for two-three days depending on the month of the year. The design used for the traps follow the model of double bottle baited with pig kidney [5], [22]. Traps consisted of plastic bottles of 1.5 l hung on trees or bushes with the bait placed on the bottom of the bottle, close to a small opening that allowed the entrance of blowflies attracted to the bait. Blowflies accessing the trap were retained on a double funnel made with two upper parts of two bottles of similar size, and later collected by the researchers. Pig kidney was selected as bait because of its similarities with human one and its great attraction power [24], [25], [26]. It was supplied by official licensed retailers, following Spanish regulations for animal by-products [27], and therefore do not fall under the remit of the Institutional Animal Care and Use Committee (IACUC). Traps were set once every month during a natural year (from July 2007 to June 2008), and every second-third day, we revisited the places and collected traps and samples. In some winter months (December−February) traps were kept active for at least three days to compensate the reduced insect activity and ensure representation of the samples. However, to avoid possible biases as a consequence of this, data were transformed into specimens captured per trapping day previous to analyses. Captured flies were separated from larvae on the kidney (which were used for development experiments) and killed by introducing them in a freezer. Then, all the imagoes collected were preserved in ethanol 70%, and identified in the laboratory to the species level following different keys [18], [28], [29], [30], [31], [32]. All analyses discussed were conducted using and considering only adult blowflies.

### Data Analysis

Captures were summarized by species and seasons. Following common practice [1], [6], 4 seasons were considered: Winter (December, January and February), Spring (March, April and May), Summer (June, July and August), and Autumn (September, October and November). Presence and abundance of species were analyzed in different seasons and different scales. Species represented by less than 100 specimens year-round were discarded from analyses, as well as those species-seasons pairs in which the species was scarce (less than 100 individuals captured) or rare (found in less than 20 locations). This ensures the representativeness, comparability and wider applicability of our results. Seasonal diversity of blowflies was assessed using Shannon diversity index [33].

To analyse relationships between species, landscape and seasons, a Multivariate ANOVA (MANOVA) was conducted with year-round data [1]. In this analysis, landscape was defined into three different categories: urban, rural and forest. Urban were dense villages and highly urbanized areas; rural included small farmlands, meadows, pastures and crops; and, as natural were categorized autochthonous forested areas and least modified ones. Assignment of each location to a category was done in the field.

### Analysis Scales

To gain further insight in the relationships between species and habitat features, and how it might be affected by landscape composition, seasonal abundance of species was analyzed against landscape descriptors at three different scales. The smallest scale was the area within 100 meters of the trapping point (i. e. an area of 0.0314 km^2^). This scale aimed to represent local features that might influence presence/abundance of blowflies. An intermediate scale of 500 meters around the sampling point (0.79 km^2^) was also considered, representing the local landscape. Finally, an area within a 2500 meter radius of the sampling point (19.63 km^2^) was considered. This distance was set after the average distance that some blowfly species are known to travel in a day [10]. Therefore, we considered that land uses within that distance could affect the presence and abundance of blowflies in the trapping areas, given the length of the trapping period (1–3 days).

### Selection and Characterisation of Independent Variables

To describe landscape composition, 1∶10 000 digital cartography with EUNIS (European Nature Information System; http://eunis.eea.europa.eu/) land use categories was used. GPS data of trapping points were uploaded into a Geographic Information System (GIS, gvGIS: http://www.gvsig.org) and three radii of 100, 500 and 2500 m were built around them. In order to minimise spatial pseudoreplication, when areas around sampling points overlapped we discarded one of them. The area covered by each EUNIS class was measured in each case and merged onto three categories: Urban, Rural and Forest. Urban included high and low density built areas, industrial areas, urban parks and gardens, roads, railways and associated infrastructures, together with harbours and other artificial areas. Rural included crops, meadows and any other areas for food production managed, including recently abandoned croplands. Finally, Forest category included all kind of forested lands of any age, including native forest and exotic tree cultures. These 3 categories encompassed more than 90% of the landscape. The remaining 10%, which included mainly sea and other water bodies, as well as rare ecosystems with scarce representation in the territory, was not considered for analysis. In addition, the following variables describing sampling points were also considered: Altitude, referred to the altitude above sea level of the exact point where the trap was placed; Distance to urban area, meaning the distance from the sampling point to the nearest densely built up area; Fragmentation, the number of different land use polygons within the considered area, as an indicator of the degree of mosaicism of the area [34], [35]; and X and Y UTM coordinates, to control possible geographic effects. The values for all these variables were calculated for each of the sampling points with the aid of the GIS using available digital cartography and digital model terrains. To detect possible interactions between predictor variables and to consider their effect on results, we built a correlation matrix of the predictor variables using Pearson’s product-moment correlation [33].

To enlighten the relationships between seasonal abundance of different species, we first estimated the degree of correlation in the abundance of pairs of species. Strong negative correlations could be interpreted as competitive exclusion and would require to be considered in further analyses, whereas lack of strong negative correlation would allow to species by species analyses. To investigate correlation we used Pearson’s product-moment correlation [33]. To analyse relationship between species abundance and landscape descriptors, we used Generalized Linear Models (GLM), which is a generalization of common linear regression that allows for several distribution functions on the response variables [11]. The GLM allows the response variable to be related to the predictor via a link function, and allows the variance to be a function of its predicted value. In our cause we performed GLMs with a Poisson error structure, fit for count data, using a logarithmic link function. We inspected the dispersion parameters of the model and their relation to the degrees of freedom looking for overdispersion [11], [36]; and when required, we accounted for it using quasi-poisson error structures [11] using the “stats” package implemented in R [37]. We only evaluated GLMs for species-season combinations for which we captured at least 100 specimens. To assess how the data fits the model we used explained deviance method, which analyses the amount residual deviance of the model against the deviance of the null model [11], [36].

All the statistical analyses were conducted using R 2.9 [37], and *p* values inferior to 0.05 were considered statistically significant in every case.

## Supporting Information

Table S1 **Correlation among descriptors of the sampling points at the three scales used.**
(DOC)
