# Supplementary material for: Seasonal Blowfly Distribution and Abundance in Fragmented Landscapes. Is It Useful in Forensic Inference about Where a Corpse Has Been Decaying?
Source: PLoS One. 2014 Jun 11;9(6):e99668. doi: 10.1371/journal.pone.0099668 (PMC4053378; doi:10.1371/journal.pone.0099668)
Supplement: Table S1 — Correlation among descriptors of the sampling points at the three scales used. (DOC) [file pone.0099668.s001.doc]

Table S1. Correlation among descriptors of the sampling points at the three scales used. We show the Pearson’s product-moment correlation coefficient, ranging from -1 (strong negative correlation) to +1 (strong positive correlation). Strong correlations (more than ±0.5) are marked in bold.

| **100m** | Rural | Urban | Altitude | | UTM Y | | UTM X | | Fragmentation | | Distance to urban | |
| --- | --- | --- | --- | --- | --- | --- | --- | --- | --- | --- | --- | --- |
| Forest | -0.297 | **-0.655** | 0.212 | 0.202 | | -0.071 | | 0.150 | | **0.508** | |  |
| Rural |  | **-0.523** | 0.038 | -0.104 | | -0.051 | | 0.048 | | 0.091 | |  |
| Urban |  |  | -0.210 | -0.107 | | 0.102 | | -0.182 | | -0.520 | |  |
| Altitude |  |  |  | **-0.744** | | -0.089 | | 0.049 | | 0.471 | |  |
| UTM Y |  |  |  |  | | 0.000 | | -0.001 | | -0.004 | |  |
| UTM X |  |  |  |  | |  | | 0.112 | | -0.178 | |  |
| Fragmentation |  |  |  |  | |  | |  | | -0.022 | |  |
| **500m** | Rural | Urban | Altitude | UTM Y | | UTM X | | Fragmentation | | Distance to urban | |  |
| Forest | -0.334 | -0.412 | 0.163 | 0.231 | | -0.042 | | 0.385 | | **0.610** | |  |
| Rural |  | -0.107 | 0.286 | -0.387 | | -0.226 | | 0.122 | | 0.016 | |  |
| Urban |  |  | -0.174 | 0.042 | | 0.196 | | 0.124 | | -0.267 | |  |
| Altitude |  |  |  | **-0.768** | | -0.088 | | 0.030 | | 0.445 | |  |
| UTM Y |  |  |  |  | | -0.007 | | 0.110 | | -0.021 | |  |
| UTM X |  |  |  |  | |  | | -0.035 | | -0.103 | |  |
| Fragmentation |  |  |  |  | |  | |  | | 0.130 | |  |
| **2500m** | Rural | Urban | Altitude | UTM Y | | UTM X | | Fragmentation | | Distance to urban | |  |
| Forest | **-0.723** | -0.446 | -0.001 | 0.446 | | -0.079 | | 0.282 | | **0.541** | |  |
| Rural |  | -0.174 | 0.291 | -0.588 | | -0.123 | | -0.160 | | -0.263 | |  |
| Urban |  |  | -0.318 | 0.091 | | 0.199 | | -0.120 | | -0.384 | |  |
| Altitude |  |  |  | **-0.762** | | -0.129 | | -0.481 | | 0.441 | |  |
| UTM Y |  |  |  |  | | 0.005 | | 0.407 | | -0.042 | |  |
| UTM X |  |  |  |  | |  | | 0.331 | | -0.067 | |  |
| Fragmentation |  |  |  |  | |  | |  | | -0.225 | |  |
